# Mapping the Published Evidence on Childhood Obesity Prevalence and Related Policies in Greece: A Scoping Review

**DOI:** 10.3390/nu17142301

**Published:** 2025-07-12

**Authors:** Raffaella Sibilio, Christos Triantafyllou, Tania Cardona, Joao Breda, Giancarlo Icardi

**Affiliations:** 1 Dipartimento di Scienze Della Salute (DISSAL), University of Genoa, Via Pastore 1, 16132 Genova, Italy; icardi@unige.it; 2 WHO Athens Quality of Care and Patient Safety Office, Ploutarchou 3, 10675 Athens, Greece; triantafyllouc@who.int (C.T.); rodriguesdasilvabred@who.int (J.B.); 3 Ministry for Health, 15, Palazzo Castellania, Merchants Street, VLT1171 Valletta, Malta

**Keywords:** childhood obesity, Greece, Southern Europe, prevalence, public health policies, obesity prevention policies, modifiable risk factors, dietary awareness

## Abstract

**Background/Objectives**: Childhood obesity is a global epidemic. Addressing the modifiable risk factors with effective policies is crucial for both prevention and intervention. This scoping review aims to provide a situational analysis of childhood obesity in Greece by mapping the available evidence on the prevalence of obesity among Greek children and adolescents and exploring the existing policies implemented to address this issue. **Methods**: A systematic literature search was conducted on 15 September 2023, using the PubMed, Scopus, and IATROTEK-online databases to identify studies related to childhood obesity and policies in Greece. Keyword groups were developed for “childhood obesity,” “Greece,” and either “prevalence” or “policies”. Additional sources, including Google and Google Scholar, were screened to ensure comprehensiveness. **Results**: A total of 66 studies were included: 61 on obesity prevalence (≤18 years of age) and 5 on existing policies tackling childhood obesity, all in Greece. The collective prevalence was observed to lie within the subsequent range of values: 2.8–21.2%. Regarding both genders, the observed prevalence ranged from 2.8% to 26.7% in males, and between 1.3% and 33.7% in females. The policies adopted in Greece cover various domains (healthy nutrition, public preferences, physical activity, school policies, and programs related to childhood obesity). **Conclusions**: Childhood obesity in Greece is a major challenge. Greece currently uses some policies and strategies to combat childhood obesity. There is still work to be done: policies play a pivotal role as a key tool to influence lifestyle habits on a broad scale and exert a considerable impact on the reduction in this prevalent health concern.

## 1. Introduction

### 1.1. Background

Childhood obesity is a global epidemic, with all countries worldwide reporting concerning prevalence rates of overweight and obese children that have increased over the past four decades, mirroring the trends observed in adults [1]. Genetic and behavioral factors that are influenced by an obesogenic environment are implicated in its etiology, with low rates of physical activity, an increased sedentary lifestyle, and unhealthy dietary habits that result in a positive energy imbalance recognized as the main culprits in children [2,3].

Child and adolescent obesity impact the quality of life and mental health during childhood. Several studies carried out in Norway [4], Germany [5,6], the Netherlands [7], Ireland [8], Australia [9], the USA [10,11], and Israel [12], among others, have proven that health-related quality of life diminished in children and adolescents with overweight and obesity, with excessive weight affecting both physical wellbeing, mental wellbeing, and self-perception [4]. Young people with overweight and obesity often experience weight-related stigma and bullying that impacts their self-esteem, which may lead to social isolation, eating disorders, body dissatisfaction, anxiety, and depression [4,5,6]. Concerns about overweight and obese children and adolescents’ health-related quality of life were also echoed by parents, with observed variations attributed to different perceptions of quality of life between guardians and their children [11,12,13].

Obesity during childhood is likely to continue into adulthood, as young people are likely to remain exposed to the same obesogenic environments as they grow older and persist in unhealthy lifestyle habits established during childhood that increase their risk of excess weight [14,15]. The prolonged exposure to the negative impacts of increased fat deposition was associated with an earlier onset of chronic diseases [1,14] and excess morbidity in early adulthood [15]. Early occurrences of diabetes, cardiovascular disease, and certain forms of cancer in both sexes were linked to childhood obesity in a systematic review [15]. Several studies also associated excessive weight in childhood with increased morbidity and mortality in the working population, leading to significant social, economic, and health implications for the individual concerned and the country [1,2,14,15]. The additional increased healthcare cost implicated for overweight and obesity can be significant, with a systematic review and meta-analysis published in 2022 indicating an increase in total annual medical expenses of USD 237.55 per capita. This study reported that non-hospital care increased by USD 56.54, outpatient visit costs by USD 14.27, medication costs by USD 46.38, and hospitalization costs by USD 1975.06 per capita. Additionally, the length of hospital stays increased by 0.28 days. Cost projections for 2050 showed an annual direct cost of USD 13.62 billion and USD 49.02 billion in annual indirect costs [16].

The severe consequences of childhood overweight and obesity have prompted policymakers to implement various actions to address childhood obesity. In the “Report of the Commission on Ending Childhood Obesity”, the World Health Organization (WHO) identified three critical life-course periods where action is needed to address the childhood obesity epidemic: preconception and pregnancy, infancy and early childhood, and adolescence. The report acknowledged the significant impact of maternal health and lifestyle choices in shaping the future risk for excess weight in children, in addition to the influence of the obesogenic environment and genetic predispositions. In particular, maternal conditions such as entering pregnancy with obesity or pre-existing diabetes, or developing gestational diabetes, predispose the child to increased fat accumulation and a higher risk of metabolic diseases and obesity later in life. The recommendations included actions aimed at improving the nutritional value of food and beverages consumed by children and adolescents all through their life course, including from preconception to during pregnancy, early infancy, childhood, and adolescence; increasing the amount of physical exercise carried out at home, school, and during leisure time; creating healthy environments that promote a healthy life; and providing evidence-based weight management programs [17]. Actions implemented in countries included legislation and regulations, recommendations and guidelines targeting the whole population, and others that focused on selective sections of the population, such as schoolchildren, as well as pregnant and lactating women [17,18].

### 1.2. Status of Obesity in Europe

Worldwide trends in child and adolescent obesity have increased across all ages, with the doubling of rates in those aged 2–4 and an 8-fold increase in those aged 5–19 years. Progress was heterogeneous amongst countries, with some higher-income countries reporting a plateau, while lower-income countries showed upward trends [19]. Within the WHO European Region, surveillance data from several national surveys including WHO’s Global Health Observatory [20], the Childhood Obesity Surveillance Initiative (COSI) [21], and Health Behaviour in School-aged Children (HBSC) [3], consistently found an increased prevalence of childhood overweight and obesity in countries in Southern Europe and from around the Mediterranean Region. The latest COSI survey carried out between 2018 and 2020 among 7–9-year-old children found that 29% of children in the WHO European Region have excess weight, with 31% of boys and 28% of girls affected. Insights from the evidence revealed that 43% of children spent at least 2 hours per day interacting with electronic devices or watching TV, while 22% consumed soft drinks on more than 3 days during the week [22]. The HBSC survey assesses 11-, 13-, and 15-year-old adolescents, with the latest findings showing that 20% of adolescents have excessive weight. Boys and younger participants had higher weight, while a third of the participating countries and regions reported an increase in rates compared to previous years [21].

A review of the literature found studies from most southern European countries, including Portugal [23,24,25], Spain [26,27], France [28], Italy [29,30], Malta [31], Croatia [32,33], Montenegro [33], Romania [34], Hungary [35,36], Serbia [37], North Macedonia [38], Cyprus [39,40], Turkey [41,42,43], and Israel [44], which provided an insight into the prevalence of childhood obesity in different study cohorts and the factors contributing to excess weight while also providing a picture of trends over time in different age cohorts and population groups.

The prevalence of excessive weight among children and adolescents increasedover the years among all age groups, with some studies indicating exponential increments [26,44] despite implementing targeted actions [26]. Other countries reported encouraging results with overweight and obesity rates levelling off in Hungary [35], as well as declining trends in some, or most, cohorts in Cyprus [40], Portugal [24], and Croatia [32]. Improvements in rates were attributed to the successful implementation of legislations and regulations finalized at improving the nutritional value of food consumed by children and an increase in the amount of physical activity carried out [24].

Most studies reviewed reported a higher prevalence of both overweight and obesity in boys [29,31,33,35,40,41], while two studies reported higher rates in girls [23,27]. On the other hand, trends in different age groups indicated that childhood obesity tends to increase with increasing age from birth, reaching a peak around ages 7–10 years and decreasing thereafter [31,36,38], with this observation attributed to the effects of puberty [38]. Different factors were found to contribute to obesity. Lifestyle choices, especially the sort of food and beverages consumed, and the level of physical activity and inactive time were investigated in several studies [29,30,31,41,45,46]. The effect of socio-economic disparities and cultural differences on overweight and obesity in children was also investigated, with foreign nationality [27], poverty and social deprivation [28,43], and low maternal educational attainment [28] found to contribute to excess weight.

The majority of studies found that overweight rates were higher than obesity in both boys and girls [23,24,28,30,39,40]; however, obesity rates were found to be on the rise, with a study in Israel reporting a 4-fold increment in obesity and a 20-fold increment in severe obesity over the past 10 years [44]. On the other hand, studies carried out in Malta [31] and North Macedonia [38] indicated that obesity rates had surpassed the prevalence of overweight. Another study from Romania reported similar findings in children who were seen in the emergency department for other reasons [34]. Studies from Portugal [23] and Italy [29] highlighted the need to monitor central obesity as a marker of visceral adiposity in addition to the BMI. These studies reported that increased risks for cardiovascular disease, diabetes, and other metabolic disorders later in life were associated with the presence of visceral fat, even when the BMI is within normal limits [23,29]. Controlling the childhood obesity epidemic is necessary, not only to prevent the short-term effects of excessive weight, such as hypertension in childhood [34], but also to prevent the occurrence of chronic diseases in early adulthood and later in life, such as type 2 diabetes, end-stage renal failure, cancer, ischemic stroke, coronary heart disease, and all-cause mortality [44].

Despite the wealth of accessible data about the prevalence of child and adolescent overweight and obesity in southern European countries, comparisons are not straightforward, particularly when different cut-off values are used to classify children according to their weight. Most studies used either the WHO cut-off values or the IOTF classification, and in some cases both measures were used. Despite these differences, childhood obesity affects all countries and requires comprehensive and intersectoral actions that involve all stakeholders [17].

### 1.3. Status of Obesity in Greece: Filling the Gap, Aims, and Objectives

This scoping review aims to provide a situational analysis of childhood obesity in Greece by mapping the available evidence on the prevalence of obesity among Greek children and adolescents and exploring the existing policies implemented to address this issue.

## 2. Materials and Methods

### 2.1. Literature Search Strategy

**Figure A1:**
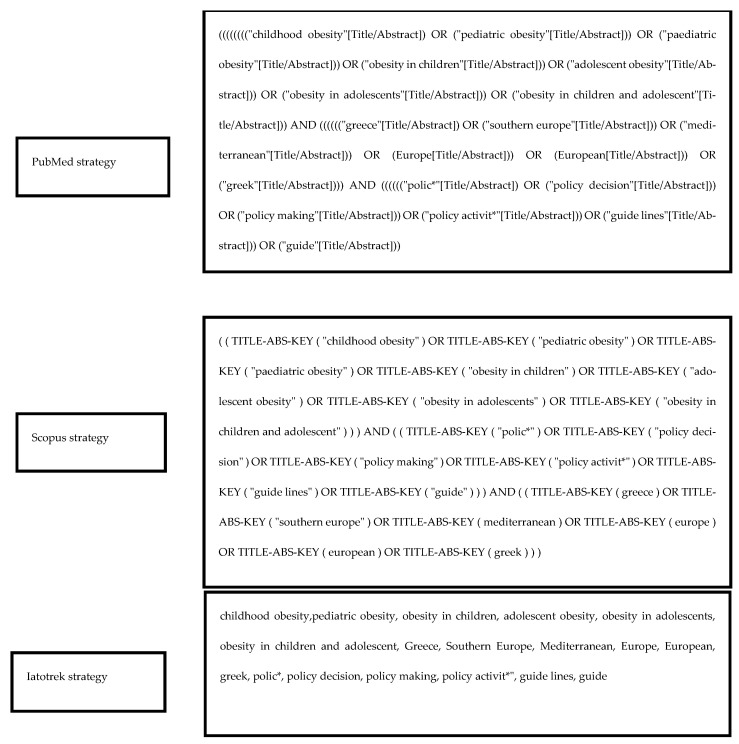
Full search strategy.

A systematic search of the PubMed, Scopus, and IATROTEK-Online databases was conducted by 1 researcher (R.S.) on 15 September 2023; no specific start date limit was intentionally applied in the search strategy, with the aim of gathering information from a wide temporal range. The search was conducted within the “title” and “abstract” fields, using three sets of keyword groups for both prevalence-related and policy-related studies; groups of terms related to ‘childhood obesity’ and ‘Greece’ were utilized in both searches, while keywords related to ‘prevalence’ were employed in the first research, and keywords related to ‘policies’ were used in the second one. The Boolean terms “AND” and “OR” were used to combine relevant terms. Appendix A presents the complete search strategy (Figure A1) used for the three databases. Cited references from chosen articles were screened to identify additional studies that were not retrieved during the initial search, while Google and Google Scholar were utilized to broaden the scope and comprehensiveness of the search.

### 2.2. Selection Criteria

Following the initial literature search, a check for duplicate studies was conducted. The remaining articles were subjected to a two-step screening process, which was performed independently by two researchers (R.S. and C.T.), to identify studies meeting the predefined inclusion criteria. In the initial step, titles and abstracts were assessed for eligibility against the predetermined criteria. Subsequently, full-text articles were evaluated when the information provided in the titles/abstracts was inadequate to make a definitive inclusion/exclusion decision. Any divergencies between the two researchers were solved through discussion with a third researcher (T.C.).

The study eligibility criteria were chosen by applying the PICOS (population, intervention, comparison, outcomes, and setting) question format. These criteria differed for prevalence and policies as follows:

1. Prevalence:
  - Population: Studies referring to children and adolescents (≤18 years of age) living in Greece were eligible; those including both adult and pediatric populations were eligible only if they presented stratified results by age group.
  - Interventions and comparators: Studies including both a group of children with obesity and without obesity were eligible, such as studies that were centered on the evaluation/presentation of an intervention, provided they included original data on prevalence before the implementation of the tool.
  - Outcomes: Studies providing data on childhood obesity prevalence in Greece were eligible.
  - Study design: Clinical trials and observational studies related to childhood obesity in Greece were eligible.
2. Policies:
  - Population: Studies referring to children and adolescents (≤18 years of age) living in Greece were eligible; those including both adult and pediatric populations were eligible.
  - Interventions and comparators: Studies including international comparisons among childhood obesity policies were eligible, such as studies that were centered on the evaluation/presentation of policy interventions tackling childhood obesity.
  - Outcomes: Studies evaluating the existing or upcoming policies to address obesity in Greece were eligible.
  - Study design: Observational studies related to childhood obesity policies in Greece were eligible.

Lastly, only studies with the full text available and published in English or Greek were considered.

### 2.3. Data Extraction

Data extraction was performed by a single researcher (R.S.), and the information was recorded in Microsoft Excel tables (Microsoft, Redmond, WA, USA). The data collected included the following: author, year of publication, country, study design, population, purpose, obesity definition used (only for prevalence studies), and key results. Regarding the prevalence, studies focusing on the same population were excluded, retaining the study with the larger sample; studies reporting a combined prevalence of overweight and obesity were not included.

## 3. Results

### 3.1. Study Selection and Characteristics

**Figure 1:**
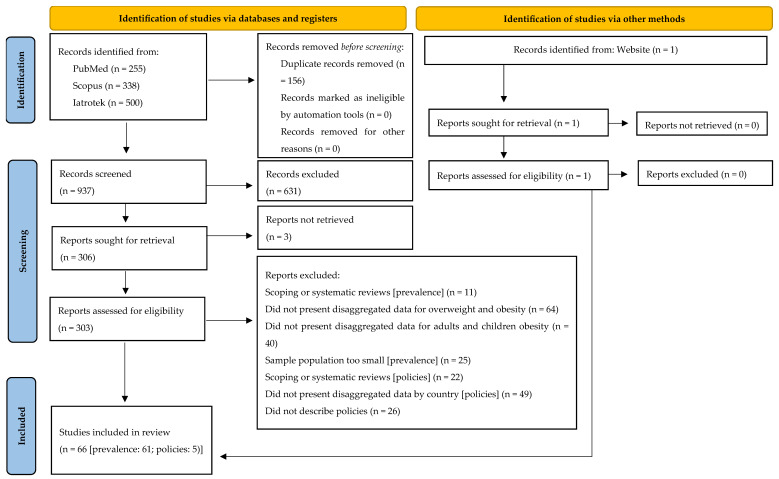
PRISMA 2020 flow diagram for new systematic reviews which included searches in databases, registers, and other sources (source: Page MJ et al. [113]).

The literature search identified 1094 studies (255 from PubMed, 338 from Scopus, 500 from Iatrotek, and 1 from other sources). After removing duplicates, 937 titles and abstracts were screened, resulting in the exclusion of 634 articles that did not meet the inclusion criteria. Full-text assessments were conducted for the remaining 303 studies, and an additional 237 studies were excluded for failing to meet the inclusion criteria. As a result, 66 studies were included in the scoping review: 61 addressed the prevalence [47,48,49,50,51,52,53,54,55,56,57,58,59,60,61,62,63,64,65,66,67,68,69,70,71,72,73,74,75,76,77,78,79,80,81,82,83,84,85,86,87,88,89,90,91,92,93,94,95,96,97,98,99,100,101,102,103,104,105,106,107], and 5 examined the policies [108,109,110,111,112]. Figure 1 shows the flow diagram of the included studies.

The majority of studies were cross-sectional (*n* = 53, 81.5%) [47,50,51,52,53,54,55,56,57,58,61,63,64,65,66,67,68,69,70,71,72,74,75,76,77,78,79,80,81,82,83,84,85,86,87,88,89,90,92,93,95,96,97,98,99,100,101,102,103,104,105,106,107]. Six studies (9.2%) were characterized as descriptive [48,60,108,109,110,111], four studies (6.2%) as epidemiological [49,62,73,91], and two (3.1%) as longitudinal [59,94]. In addition, a document published on the official UNICEF webpage was also included as it was deemed relevant for the purposes of the review.

The majority of studies used to assess the prevalence of childhood obesity in Greece (*n* = 37, 60.7%) were conducted on student populations attending primary schools [47,50,53,54,56,57,61,62,63,65,67,68,69,71,72,76,77,78,79,80,81,82,84,85,88,89,90,91,95,96,97,99,100,101,104,105,106]; out of the 41 studies (67.2%) that reported gender-specific data, 23 (56.1%) indicated a predominance of females [48,49,51,53,54,60,61,62,63,64,68,69,74,75,77,80,84,88,96,98,101,105,106]. Furthermore, 51 (83.6%) applied the International Obesity Task Force [IOTF] criteria to define obesity [47,49,50,52,53,54,56,58,59,60,61,63,64,65,68,69,70,71,72,73,74,75,76,77,79,80,81,82,83,84,85,86,87,90,92,93,94,95,96,97,98,99,100,101,102,103,104,105,106].

Among the articles included in the scoping review, the earliest publication is from 2001 [67], while the most recent ones were published in 2023 [48,74,75,110].

### 3.2. Prevalence

From the analysis of the included studies on prevalence (Table 1), it is worth noting that the highest observed value was 57.1% [mean age (±SD) of 15.09 ± 1.81 years], followed by 25.1% [mean age (±SD) of 15.09 ± 1.81] in studies that focused on populations attending healthcare services for the control of overweight and obesity [49,60]; the highest value for obesity prevalence in studies involving the general pediatric population was 21.2% [mean age (±SD) of 13.6 ± 0.9 years] [95]. With respect to the lowest prevalence rate, it was observed to be 2.8% [85]; however, in a study which presented results stratified by age, the lowest prevalence rate detected was 1%, referring to the population with the age of two years [94]. Regarding both genders, the highest prevalence rates observed were 66.3% in males and 49.5% in females [mean age of both sexes (±SD) of 10.10 ± 0.09 years], in a study targeting populations attending clinics for the control of overweight and obesity [60]. In studies referring to the general population, the highest prevalence observed was 26.7% in males [mean age (±SD) of 9.1 ± 1.8 years], while in females, it was 33.7% [mean age (±SD) of 9.3 ± 1.8 years] [50]. The lowest prevalence reported for males was 2.8% [mean age 7.9] [85], while for females, it was 1.3% [mean age (±SD) of 12.4 ± 1.5 years] [83].

Direct comparisons between studies were not possible due to differences in the time of data collection, the methodology, and the target population.

### 3.3. Policies

According to the included studies on policies (Table 2), Greece presently enforces a multifaceted policy framework designed to combat childhood obesity, incorporating various strategic dimensions: school-centric measures focus on the school environment, involving the oversight of food provided in school cafeterias, curbing the presence of obesogenic foods and related advertisements [111]; family-oriented strategies entail educational initiatives targeting children and parents in matters of nutrition and dietary conducts [108], and soliciting public opinions on preferred anti-obesity policy directions plays a pivotal role [109]; community-wide interventions are instrumental in creating environments conducive to physical activity, extending beyond school boundaries, and include workforce training within healthcare facilities and community health organizations [110]; healthcare service provisions aim to heighten awareness among pediatric healthcare practitioners about childhood obesity issues and advocate for enhanced collaboration between educational institutions and healthcare professionals to ensure comprehensive follow-up care for children and adolescents grappling with obesity [112]; the research component also assumes a fundamental position, contributing to the expansion of knowledge and the formulation of evidence-based policy recommendations [112].

## 4. Discussion

The primary aim of this scoping review is twofold: first, to provide a detailed analysis of the prevalence of childhood obesity in Greece in order to gain an accurate understanding of the extent of the issue faced by the country and second, to critically analyze the existing policies addressing this problem, with the goal of identifying points of implementation and potential areas for improvement. To combat childhood obesity, indeed, it is crucial to comprehend both its prevalence and the existence of national strategies to tackle it, as these measures furnish us with the tools and approaches to confront this pressing public health issue. What makes this study unique is its approach of merging the examination of childhood obesity prevalence in Greece with a detailed investigation of the preventive policies implemented in the country; until now, previous research and analyses have primarily focused on one of these aspects, often overlooking the opportunity to fully understand the interplay between the epidemiological situation and government initiatives. Our scoping review aims to bridge this gap by providing a comprehensive overview of the situation in Greece, offering a different perspective on the challenge of childhood obesity; through this innovative combination of data and analysis, we aim to lay the groundwork for a deeper understanding and significant progress in managing childhood obesity, not only in Greece but also on an international scale.

The overall prevalence exhibited a range of values from 2.8% [mean age of 7.9 years] [85] to 21.2% [mean age of 13.6 years] [95]. In terms of gender-specific prevalence, the observed rates ranged from 2.8% [mean age 7.9 years] [85] to 26.7% [mean age of 9.1 years] in males [50] and from 1.3% [mean age of 12.4 years] [83] to 33.7% [mean age of 9.3 years] in females [50]. The results highlighted a significant variation in the prevalence of childhood obesity in Greece, both in terms of overall values and distribution between genders. Most of the studies included in this review were cross-sectional (53 out of 61), with very few epidemiological, longitudinal, or descriptive studies. This predominance limits the ability to assess causality and trends over time and makes the findings more susceptible to recall and selection bias.

To provide context for these results on the prevalence of childhood obesity, it is pertinent to make some observations about the European scenario into which Greece fits.

According to data derived from the fifth round of the WHO COSI surveillance, the prevalence of childhood obesity, as defined by the WHO criteria, exhibited significant disparities across European countries. Notably, Cyprus reported the highest prevalence, standing at 19%, followed by Greece, (which also displayed a rate slightly less than 19%) ranking alongside Italy; these results confirm the overweight–obesity gradient, whereby these conditions have a higher prevalence in Southern European countries [21]. In contrast, Tajikistan, Denmark, Kazakhstan, and Israel reported much lower prevalence rates [21]. When the IOTF criteria are applied to assess obesity prevalence in Greece, the numbers continue to be noteworthy. Among 7-year-olds, the prevalence of obesity stood at 11.7% for boys and 10.1% for girls; furthermore, 9-year-old boys and girls showed rates of 10.0% and 6.8%, respectively [21]. It is intriguing to observe that Greece is among the select group of countries that witnessed a decline in childhood obesity prevalence between the first (2007–2008) and fourth (2015–2017) rounds of the COSI surveillance—a group that also includes Italy, Slovenia, Portugal, and Spain. However, when comparing obesity prevalence between the fifth round and the previous cycle (2015–2017), statistically significant changes were limited to an increase among boys in Georgia and decreases among boys in San Marino and girls in Malta [21].

These findings highlight the diverse patterns of childhood obesity prevalence across Europe, with Greece holding a notable position in the context; the data from the WHO surveillance reveals that the situation in Greece is not isolated but is part of a broader European context where childhood obesity presents a significant challenge. These results align with the fact that Greece is a part of Southern Europe, and as such, it is in accordance with this prevailing trend. It is indeed a positive outcome that the prevalence of childhood obesity in Greece, along with other countries, exhibited a reduction between the first and fourth rounds of COSI [21]; nonetheless, given the statistically significant increase in obesity prevalence among girls in Georgia between the fourth and fifth rounds of COSI, the importance of strengthening prevention efforts cannot be overstated [21].

Regarding policies to combat childhood obesity, Greece is currently enacting strategies that encompass the application of the “Greek Nutrition and Diet Guidelines” (NDGGr), which provide recommendations for a balanced diet and promote key messages on healthy dietary patterns and lifestyle habits; the involvement of the Greek public in determining the most important and effective policies to establish a healthier diet for the pediatric population; the existence of physical activity policies in Greece, targeting schools, communities, workplaces, and healthcare facilities; the successful implementation of the “Greek School Canteen Policy,” effectively limiting the presence of unhealthy foods in the school environment and imposing restrictions on advertising unhealthy foods within educational institutions; and the promotion of programs to prevent childhood obesity in Greece through the “Childhood Obesity Prevention Program,” involving health centers, schools, and the community.

The nutrition guidelines are considered a fundamental policy not only for combating childhood obesity but also for addressing any form of malnutrition; they are also deemed essential for promoting a healthy lifestyle. For these reasons, among the data collected for the tracking and oversight of childhood obesity, the WHO Regional Office for Europe has made available the e-Library of Evidence for Nutrition Action (eLena) since 2011; eLENA serves as a digital repository housing evidence-based guidelines for a continually growing array of nutritional interventions [117]. This resource functions as a centralized hub that offers the most up-to-date dietary guidelines, advice, and related resources, which encompass empirical evidence endorsing these directives, as well as statements providing biological, behavioral, and contextual rationale; furthermore, it provides observations contributed by recognized experts in the field. eLENA’s overarching objective is to support nations in the effective implementation and expansion of nutritional interventions. It achieves this by serving as an informative resource and guiding the development of policies and the design of programs [117]. Greece, starting in 2019, has embraced the national nutrition guidelines as one of the cornerstones in the fight against childhood obesity, much like the majority of other WHO European countries. It is important to emphasize that despite variations in geographical, socio-economic, and cultural contexts across countries, most of the essential nutritional recommendations remain consistent [108]. These key messages advocate for the everyday consumption of sufficient quantities of fruits, vegetables, and dairy products, as well as starches, cereals, and grains, along with a moderate-to-limited intake of fats. Additionally, it is worth noting that a majority of the countries within the WHO European Region provide guidelines on physical activity, but just a few recommend physical exercise for at least 60 min per day, which aligns with the highest recommendation, mirroring the approach taken by the NDGGr [108].

When it comes to considering public opinion, it is important to mention that assessing public perspectives on health policies can yield practical advantages [109]. This includes pinpointing areas where knowledge gaps or misunderstandings exist regarding suitable health behaviors. Moreover, it allows us to measure various factors associated with health policy opinions, such as gender, race, and education. In countries like the United States, Germany, and Australia, previous scientific investigations have delved into gauging public support for government interventions related to obesity, identifying factors linked to specific policy endorsements, and tracking shifts in public sentiment over time [109]. The European Union [EU] public generally supports specific policies aimed at addressing childhood obesity, which include promoting communication with parents, delivering healthy nutrition education to children, and increasing physical activity within school settings [109]. Limited endorsement was evident for certain policy strategies that have frequently been employed by policymakers: enhancements to school meal programs and levies on unhealthy nutritional products were preferred only by a small minority of the participants; this could serve as a valuable point of reflection to better understand how to proceed with the implementation of future policies [109]. The Greek public’s preferences regarding policy strategies to combat childhood obesity align with those of the EU public [109]: they prioritize improving children’s diets, with a significant emphasis on providing information to parents. Additionally, Greek citizens prioritize physical activity programs in schools, education on diet and exercise, and further restrictions on food advertisements. It is noteworthy that Greece has closely aligned itself with countries (Cyprus, Greece, Italy, Malta, Portugal, Spain, and Turkey) in its region (Southern Europe) in terms of supporting the parental information policy. On the other hand, it has diverged from its regional counterparts concerning support for physical activity [109]: the public in other southern nations did not rank physical activity as one of their preferred policies; in contrast, the highest support was observed in Southeastern European countries (Croatia and Slovenia).

To provide context for the physical activity policies adopted in Greece, it is essential to consider that European countries have been active in implementing various policy actions related to physical activity. These include educational policies focusing on physical activity programs in schools and communities, environmental policies creating physical activity-friendly environments like cycling lanes and parks, fiscal policies providing subsidies for sports and recreational activities, and marketing policies aimed at promoting physical activity through campaigns and awareness programs [117]. Substantial progress has been made, but further investments and coordination are needed. Countries that have embraced coordinated, multisectoral approaches and developed supportive environments for physical activity, such as Ireland, the United Kingdom, and Finland, are more likely to achieve better outcomes in the fight against childhood obesity [110]. Greece has already implemented several physical activity policies, encompassing educational programs, environmental enhancements, and fiscal support for sports and recreational activities [110], but it is actively seeking areas for improvement: for instance, physical activity education programs in Greek schools often suffer from insufficient funding; furthermore, the Greek environment does not always facilitate physical activity, with a limited presence of cycling lanes and parks. However, Greece’s commitment to tackling these issues demonstrates a proactive approach and a willingness to progress further.

National school nutrition policies have been adopted by all 28 EU Member States [117]. Approximately half of these nations have instituted mandatory standards, while the other half have provided voluntary guidelines. These policies exhibit a wide range of approaches in their development, from simple lists of permitted or prohibited nutrients within schools, as seen in Cyprus and Greece, to comprehensive sets of instructions addressing various elements of dietary regulation such as procurement, food provision services, and meal preparation and dining spaces, as observed in France and Spain [117]. The primary goals of these policies are to enhance child nutrition, promote healthy eating habits and lifestyles among children, and combat or prevent childhood obesity. These objectives are shared by a significant percentage of the policies, with 97% focusing on improving child nutrition, 94% emphasizing healthy eating habits and lifestyles, and 88% targeting the reduction or prevention of childhood obesity [117]. Regarding the “Greek School Canteen Policy,” it should be noted that it has evolved through collaborative efforts, effective communication, and vigilant monitoring procedures. Certain countries, including Greece, Latvia, and Hungary, have instituted formal monitoring mechanisms to oversee the enforcement of specific legislations; in the case of Greece, a comprehensive control system has been put in place to assess the adherence of school canteens to established policies [111]: this oversight involves public health supervisors from each prefecture and certified auditors from the Hellenic Food Authority (EFET). Inspection evaluations are meticulously documented, with a specific form used to determine compliance with valid criteria. At the commencement of every new school year, the Ministry of Health proactively reminds prefectures to initiate investigations in all available schools. Inspections are thoughtfully planned on an unpredictable schedule and may be carried out either with or without prior notice to the head of the school, including in response to complaints regarding the products available in school canteens or dining areas. The Ministry of Health maintains a comprehensive list that explicitly outlines the permissible products in school canteens, ensuring a clear and unambiguous framework [111].

The Childhood Obesity Prevention Program, jointly developed by the Greek Ministry of Health and UNICEF, is a comprehensive action plan aimed at addressing all areas that can promote the prevention and control of childhood obesity [112]. This program encompasses initiatives spanning primary, secondary, and tertiary prevention, focusing on children aged 0–17 and their families. Its primary objective is to address risk factors and socio-economic disparities associated with childhood and adolescent obesity while also combatting the long-term health implications frequently associated with excess weight during adulthood [112]. Greece is not the only nation to have embraced the idea of creating an integrated plan to combat childhood obesity while also addressing health inequalities, identified as a key factor influencing obesity development. Also Spain has developed the “Plan Estratégico Nacional para la Reducción de la Obesidad Infantil”, which was adopted in 2022. This plan was created and developed by the Spanish government in close collaboration with UNICEF and the WHO. Its purpose [118] is to reduce childhood obesity by 25% over the next decade, focusing on physical activity, healthy nutrition, adequate rest, and emotional wellbeing; it adopts a child rights-based approach, with measures impacting all aspects of a child’s life. The social determinants of health approach, a conceptual framework developed by the WHO, emphasize that the social, cultural, and environmental conditions in which people live with substantially influence their lifestyles and health [118]. The Greek action plan closely aligns with the Spanish plan in numerous key aspects [112]: to address childhood obesity, it recommends implementing comprehensive and intersectoral measures that integrate the links between healthy individuals, healthy societies, and healthy environments; also, the plan is implemented through an action framework in the main environments where children and adolescents live and grow: the family, educational, healthcare, active leisure and sports, urban (towns and cities), digital, and audiovisual environments, as well as the macrosocial environment, which spans across all the others.

Overall, Greece has implemented a range of policies and programs to address childhood obesity and promote healthy lifestyles; although there are areas for improvement, these policies reflect the country’s commitment to tackle this public health issue.

This study is subject to several inherent limitations. Firstly, it is prudent to acknowledge the potential presence of language bias, as the review exclusively incorporated studies composed in English and Greek. The decision to restrict inclusion to these languages was prompted by practical constraints, particularly the challenges associated with translating content from a multitude of languages. Furthermore, the search strategy’s exclusive reliance on electronic databases could have resulted in publication bias, as this method might overlook studies that were not published in peer-reviewed journals.

Another notable limitation is the variability in the definitions of obesity used across the included studies. As this is a descriptive review, we included all studies meeting the eligibility criteria regardless of the classification system applied, specifying the criteria used in each study in Table 1. Moreover, additional limitations relate to the heterogeneous age ranges within the pediatric population targeted by the prevalence studies, the presence of some outdated data, and the wide time span covered by the included studies, all of which should be taken into consideration. This heterogeneity limits the direct comparability of prevalence estimates across studies. In addition, this review considered overall obesity prevalence without assessing abdominal (central) obesity separately, which may have limited a more specific evaluation of health risks. Another aspect to consider is that, as a descriptive review, this study does not include a quantitative synthesis or meta-analysis, which limits its ability to provide pooled estimates or to explore potential causal relationships and trends over time. Concerning Greek policies, the foremost limitation, which also represents a significant discovery, is the paucity of published studies focusing on the subject matter. However, the limited number of studies included may lead to an incomplete representation of the policy environment, potentially overlooking existing structural efforts that are not formally documented. A further key limitation is the lack of data regarding the effects or outcomes of the implemented policies. Given the descriptive nature of this study, we focused on identifying existing policies without assessing their impact or effectiveness. Future research should address this gap by evaluating how these policies influence childhood obesity rates and related health outcomes.

## 5. Conclusions

The prevalence of childhood obesity in Greece fluctuates significantly, ranging from 2.8% to 21.2%. This variation is not only based on age but also on gender, with different rates for boys and girls. Greece currently adopts policies and strategies targeting childhood obesity, prioritizing balanced nutrition, physical activity, and public involvement. These policies place an emphasis on monitoring, coordination, and collaboration among different stakeholders. However, further efforts are needed to achieve a sustained reduction in childhood obesity rates in Greece. Enhancing surveillance systems is crucial for improving our understanding of the mechanisms underlying the development of obesity. Such intensified monitoring is essential not only to support future research but also to increase public awareness, including through collaboration with the media. Moreover, it is important to evaluate the effectiveness of existing policies in Greece and assess their efficiency, allowing for adaptation to the specific needs of the population. Further national studies are needed to investigate these aspects, including a deeper examination of the social determinants of childhood obesity, in order to provide updated evidence that can inform policy decisions and guide targeted interventions.

## Figures and Tables

**Table 1:** Prevalence study table.

Author (Year) [Ref]	Country	Study Design	Population	Purpose	Obesity Definition Used	Key Results
Grigorakis et al. (2016) [47]	Greece	Cross-sectional study	124.113 Greek children attending the third and fifth grades of primary school (mean age: 9.9 ± 1.1 years). A total of 50.8% (*n* = 63.064) were boys, and 49.2% (*n* = 61.049) were girls.	To determine the prevalence of central obesity and its association with dietary and physical activity habits in a large sample of Greek schoolchildren participating in a nationwide school-based health survey.	BMI was calculated, and International Obesity Task Force (IOTF) cut-offs were used to classify the children’s weight status.	More boys were obese compared to girls. Boys: Obesity = 9.6%. Girls: Obesity = 8.0%. Total obesity: 8.8%.
Bastida et al. (2023) [48]	Multicenter	Descriptive study (Intervention)	9–12-year-old children. Subjects = 156; female (%) = 59.6; age (years ± sd) = 10.12 ± 1.45.	The paper presents OCARIoT, a solution designed to prevent obesity onset through the promotion of healthy habits among children aged 9–12 while solving the current limitations of similar systems.	The stratification for nutritional status was based on the World Health Organization (WHO) BMI-for-age levels.	GREECE. Obesity = 5.8%.
Andrie et al. (2021) [49]	Greece (Athens)	Epidemiological Study (Case–control)	A total of 414 adolescents (who attended the tertiary “P. & A. Kyriakou” Children’s Hospital in Athens, Greece) with a mean age (±SD) of 15.09 ± 1.81 years participated in this study. Among them, 233 (56.3%) were girls, while 181 (43.7%) were boys. The control group of adolescents with normal weight was recruited from outpatient services of the same hospital.	The study aims to identify psychosocial factors associated with excess body weight of adolescents.	BMI was calculated, and International Obesity Task Force (IOTF) cut-offs were used to classify the children’s weight status.	A total of 25.1% of the enrolled adolescents were in the obese range. Boys comprised about half of the obese group.
Kollias et al. (2011) [50]	Greece (Eastern Attica, Kalivia)	Cross-sectional study	A total of 780 students (9.2 ± 1.8 years old (age range: 6–13 years)) of middle socio-economic class from all seven schools of the municipality of Kalivia were assessed: 415 boys (9.1 ± 1.8 years old) and 365 girls (9.3 ± 1.8).	To examine the prevalence and determinants of obesity and associated cardiovascular risk factors in a sample of Greek children.	These proposed cut-offs for overweight and obesity, adapted by the International Obesity Task Force (IOTF), correspond to an adult BMI of 25 and 30 kg/m2.	Approximately 8% of the participants were obese. The overall prevalence of obesity in the study population was 65 out of 780 (8.3%). The obese state was more frequent among females in comparison to males (33.7% vs. 26.7%, respectively).
Malindretos et al. (2009) [51]	Greece (Thesprotia)	Cross-sectional study	107 children aged 12.2 ± 0.78 years living in Thesprotia, Greece: 50 males and 57 females.	To estimate the prevalence of obesity in school-aged children living in one of the poorest districts of Europe, as well as to estimate the association between the frequency of obesity observed in these children and their parents.	Body mass index (BMI) measurements were adjusted for age and sex (according to NHANES I).	A total of 17 children (16%) were obese. Approximately half of them were males (eight boys), while the rest were females (nine girls).
Sergentanis et al. (2021) [52]	Multicenter	Cross-sectional study	This study analyzed 8785 adolescents aged 14–17.9 years old from the EU NET ADB survey.	This study attempts to evaluate the association between cyberbullying victimization and overweight/obesity in adolescents participating in the European Network for Addictive Behavior (EU NET ADB) survey.	BMI was calculated, and International Obesity Task Force (IOTF) cut-offs were used to classify the children’s weight status.	1516 of the adolescents enrolled in the study were from Greece; among them 49 were obese (obesity rate = 3.2%).
Athanasopoulos et al. (2011) [53]	Greece (Kalymnos)	Cross-sectional study	232 schoolchildren from two primary and two secondary public schools on the island of Kalymnos. Mean age 12.2 ± 2.6 years; 43.9% (*n* = 102) male.	To estimate the prevalence of increased weight in children and adolescents on a remote Greek island in the Aegean Sea and to assess the factors influencing this phenomenon.	BMI was calculated, and International Obesity Task Force (IOTF) cut-offs were used to classify the children’s weight status.	In total 8.1% (*n* = 19) of the participants were classified as obese.
Jelastopulu et al. (2012) [54]	Greece (Patras)	Cross-sectional study	A total of 200 children (10–13 years old) from primary schools across Patras were assessed: a total of 92 were boys, while 108 were girls; mean age = 11.2 years.	The purpose of this cross-sectional study was (i) to assess in an objective way the weight status (BMI and waist circumference [WC]) in a representative, random sample of children attending the last two grades of primary schools in an urban region; (ii) to assess the rate of overweight and obesity by sex in these age groups; and (iii) to identify the most significant associations and possible risk factors for being overweight or obese among nutritional attitudes, daily activities, and parental characteristics.	BMI was calculated, and International Obesity Task Force (IOTF) cut-offs were used to classify the children’s weight status.	The overall prevalence of obesity was 10.5%. The prevalence of obesity in boys was 13%, and in girls, it was 8.3%. Boys (*n* = 92): Obese 13% (*n* = 12)
Manios et al. (2009) [55]	Greece	Cross-sectional study	2374 children aged 1–5 years, recruited in the study from a representative sample of randomly selected public and private nurseries as well as day-care centers within municipalities in five counties of Greece.	To evaluate the effect of preschoolers’ television (TV) watching time on the prevalence of obesity even after controlling for their total energy intake and their physical activity status.	Obesity was defined using the age- and sex-specific growth charts of the Centers for Disease Control and Prevention (CDC).	A total of 17.6% of participants were obese.
Kyriazis et al. (2012) [56]	Greece	Cross-sectional study	2374 students (1206 males and 1168 females) 6 to 12 years old.	To evaluate the prevalence of overweight and obesity in children.	BMI was calculated, and International Obesity Task Force (IOTF) cut-offs were used to classify the children’s weight status.	BMI measurements showed that 7.3% of the children were classified as obese. The boys were obese in a higher percentage than the girls (9.2% vs. 5.3%). The obesity rates were gradually reduced as the children were growing older: for the 6–9-year-old group, the obesity percentage was 10.3%, while in the 10–12-year-old group, the percentage was 3.3%.
Mavrakanas et al. (2009) [57]	Greece (North)	Cross-sectional study	A total of 572 schoolchildren were examined (287 boys and 285 girls). The mean age of the children in the study was 7.3 ± 2.0 years.	To determine the prevalence of childhood obesity and elevated blood pressure (BP) in a rural population of northern Greece.	Obesity was defined using the following methods: (i) BMI charts of the French society of Paediatrics (FR97) and (ii) US95 BMI charts produced by the Centers for Disease Control in 2000.	Obesity in males: 4–5.9 years (*n* = 52): 17.3% FR97-BMI; 17.3% US95-BMI; 6–6.9 years (*n* = 85): 28.2% FR97-BMI; 20.0% US95-BMI; 8–8.9 years (*n* = 67): 37.3% FR97-BMI; 29.9% US95-BMI; 10–10.9 years (*n* = 83): 39.8% FR97-BMI; 26.5% US95-BMI; Total (n287): 31.7% FR97-BMI; 23.7% US95-BMI. Obesity in females: 4–5.9 years (*n* = 69): 17.4% FR97-BMI; 14.5% US95-BMI; 6–6.9 years (*n* = 63): 28.6% FR97-BMI; 15.9% US95-BMI; 8–8.9 years (*n* = 87): 48.3% FR97-BMI; 27.6% US95-BMI; 10–10.9 years (*n* = 66): 42.4% FR97-BMI; 24.2% US95-BMI; TOTAL (n285): 35.1% FR97-BMI; 21.1% US95-BMI.
Manios et al. (2018) [58]	Multicenter	Cross-sectional study	7554 preschool-aged children (born between January 2007 and December 2008): 51.9% boys and 48.1% girls; mean age of total sample (years ± sd): 4.7 ± 0.4.	To record the prevalence of overweight and obesity among preschoolers across six European countries in relation to sociodemographic and family factors.	BMI was calculated, and International Obesity Task Force (IOTF) cut-offs were used to classify the children’s weight status.	Prevalence of obesity among preschool-aged children in Greece: 5.7%.
Margetaki et al. (2022) [59]	Greece (Crete, Heraklion)	Longitudinal study	The sample size for growth analyses is 747 children: the first contact was made at the time of the first examination: mean age ± SD = 11.96 ± 1.49 weeks; boys = 53.7% (*n* = 401); several contacts followed (6th month of pregnancy, at birth, 9 months, 1st year, and 4 and 6 years after birth). For all subsequent analyses, the sample sizes varied depending on the availability of the outcome data. Mean age at 4 years: 4.2 ± 0.2 (*n* = 717); mean age at 6 years: 6.6 ± 0.3 (*n* = 501).	To investigate the associations of prenatal and postnatal exposure to antibiotics on childhood growth and obesity, as well as cardiovascular traits, at ages 4 and 6 years.	BMI was calculated, and International Obesity Task Force (IOTF) cut-offs were used to classify the children’s weight status.	4 years: Obesity = 7.8% (*n* = 55). 6 years: Obesity = 12.0% (*n* = 60)
Tragomalou et al. (2020) [60]	Greece	Descriptive study (Intervention)	2400 children and adolescents aged 2–18 years attending the out-patient clinic for the prevention and management of overweight and obesity in childhood and adolescence; mean age ± SEM: 10.10 ± 0.09 years; 1088 males and 1312 females.	To evaluate the effectiveness of the interventions suggested by the electronic system in reducing the prevalence of obesity and overweight and to present the progress of a large number of children and adolescents who have followed the personalized multi-disciplinary management plan specified by the ‘National e-Health Program for the Prevention and Management of Overweight and Obesity in Childhood and Adolescence’.	BMI was calculated, and International Obesity Task Force (IOTF) cut-offs were used to classify the children’s weight status.	Total: Obesity (*n* = 1370): 57.1%. Males (*n* = 1088): Obesity: 66.3%. Females (*n* = 1312): Obesity: 49.5%. A significantly higher number of boys had obesity compared to girls (66.3% vs. 49.5%). There was no significant difference in BMI category between prepubertal and pubertal children.
Hassapidou et al. (2009) [61]	Greece (Thessaloniki)	Cross-sectional study	266 schoolchildren (130 boys and 136 girls): 236 Greeks and30 immigrants. Mean Age (years ± sd): Total = 9.98 ± 0.95; Greek = 9.92 ± 0.92; immigrants = 10.40 ± 1.07.	To investigate sociodemographic, ethnic, and dietary factors associated with the development of childhood obesity.	BMI was calculated, and International Obesity Task Force (IOTF) cut-offs were used to classify the children’s weight status.	More than 11.6% of the boys and 10.9% of the girls were obese. Overall Obesity (%) = 11.7.
Tsolakis et al. (2022) [62]	Greece (regional unit of West Attica)	Epidemiological study	399 students (187 boys and 212 girls) from four schools, aged 8–12.	To investigate the relationship of body mass index (BMI) with muscle and cardiorespiratory fitness in children living within rural areas in Greece.	BMI was calculated. Students’ weight status was classified according to the BMI cut-off points of the World Health Organization (WHO) 2007 [114] norms.	The prevalence for obese boys and girls was 26.2% and 24.2%, respectively.
Kosti et al. (2022) [63]	Greece (metropolitan Athens area, Heraklion, and in three main counties of the Peloponnese peninsula: Sparta, Kalamata, and Pyrgos)	Cross-sectional study	1688 schoolchildren (45.9% boys and 54.1% girls), aged 10–12. Mean age (years ± sd) = 11.20 ± 0.78.	To examine the co-influence of breakfast eating habits, sleep duration, and physical activity on the weight status of children 10–12 years old from several schools in Greece.	BMI was calculated, and International Obesity Task Force (IOTF) cut-offs were used to classify the children’s weight status.	A total of 6.5% of the boys and 4.0% of the girls were categorized as children with obesity; mean age of children with obesity (years ± sd) = 11.11 ± 0.75.
Patsopoulou et al. (2015) [64]	Greece (Larissa)	Cross-sectional study	451 adolescent students (12–18 years old): 158 males and 293 females.	Investigating the prevalence of overweight and obesity in adolescents and their parents and identifying associated factors among parents’ and adolescents’ demographics, eating habits, and parental style.	BMI was calculated, and International Obesity Task Force (IOTF) cut-offs were used to classify the children’s weight status.	A total of 4.2% of the adolescents were obese. Obesity Rate: 8.9% for males (*n* = 14/158) and 2.0% for females (*n* = 6/293)
Farajian et al. (2011) [65]	Greece (Attica, Macedonia, Peloponnisos, Sterea Ellada and Evia, Ipeiros, Thessalia, Thrace, Aegean islands, Ionian islands, and Crete)	Cross-sectional study	4786 children, aged 10 to 12 years old.	To provide current national data on overweight and obesity prevalence in preadolescent schoolchildren (aged 10–12 years old) in Greece and, additionally, to evaluate the quality of children’s diets by assessing the degree of adherence to the Mediterranean diet and its association with the obesity rates.	BMI was calculated, and International Obesity Task Force (IOTF) cut-offs were used to classify the children’s weight status.	The overall prevalence of childhood obesity (OB) was 11.7%. The prevalence of obesity was higher in boys than girls; additionally, no differences were found between different age groups (10, 11, and 12 years old) concerning OB prevalence for both genders and the overall sample.
Kosti et al. (2007) [66]	Greece (Vyronas)	Cross-sectional study	2008 students (1021 male and 987 female), 12–17 years of age (7–12th grade).	To evaluate the dietary habits and some lifestyle characteristics of Greek adolescents 12–17 years of age in relation to the prevalence of overweight/obesity.	Overweight and obesity were defined using the international body mass index (BMI) cut-off points established for children and youths.	Overall, 4.4% of the boys and 1.7% of the girls were obese.
Krassas et al. (2011) [67]	Greece (Thessaloniki)	Cross-sectional study	2458 schoolchildren aged 6 to 17 years from 27 primary and secondary public schools; 6–10-year-old group = 1226 children; 11–17-year-old group = 1232 children.	To investigate the prevalence of overweight and obesity among children and adolescents in the city of Thessaloniki and evaluate the trends in Greece by comparing the results to those of other cross-sectional studies.	The estimations of the prevalence of overweight and obesity are based on recently established international BMI percentile curves and cut-off points from 2 to 18 years.	In the younger group (6–10 yr), the prevalence of obesity was 5.6%, while for adolescents (11–17 yr) it was 2.6%. The obesity prevalence for males was 5.1%, while for girls, it was 3.2%.
Karachaliou et al. (2020) [68]	Greece	Cross-sectional study	11,751 children from primary and secondary schools located in several municipalities in Greece. 49.3% boys (*n* = 5798); 50.7% girls (*n* = 5953). Mean age (years ± sd): 9.1 ± 1.7.	To evaluate the prevalence of asthma symptoms in a representative sample of Greek schoolchildren and to evaluate its association with overweight/obesity as well as other socio-economic, demographic, and lifestyle factors.	BMI was calculated, and International Obesity Task Force (IOTF) cutoffs were used to classify the children’s weight status.	A total of 10.5% (*n* = 1228) of the children in the study sample were obese, and 26% (*n* = 3050) were overweight.
Manios et al. (2016) [69]	Greece	Cross-sectional study (tool validation)	5946 schoolchildren and adolescents in the ages from 6 to 15 years: 50.9% females (*n* = 3024) and 49.1% males (*n* = 2922); mean age (sd): total = 11.1 years (2.7); girls = 11.1 years (2.7); boys = 11.1 years (2.7).	To examine the utility and applicability of the “Childhood Obesity Risk Evaluation (CORE)” index as a screening tool for the early prediction of obesity in childhood and adolescence.	BMI was calculated, and International Obesity Task Force (IOTF) cut-offs were used to classify the children’s weight status.	Total obesity rate = 9.1% (*n* = 543)
Moschonis et al. (2017) [70]	Multicenter	Cross-sectional study	Greek population: 309 4-year-old children. Data available for newborns between October 2005 and October 2007 in two different clinics in Athens.	To examine the association of feeding practices during infancy with growth and adiposity indices in preschool children from four European countries and in UK schoolchildren and adolescents.	BMI was calculated, and International Obesity Task Force (IOTF) cut-offs were used to classify the children’s weight status.	Obesity = approx. 6%.
Tokmakidis et al. (2006) [71]	Greece (Alexandroupolis and Agios Stefanos)	Cross-sectional study	709 schoolchildren (328 girls and 381 boys) of elementary schools; mean age (years ± sd) = 8.9 ± 1.6.	To provide estimates for overweight and obesity in a sample of Greek schoolchildren and to determine their possible relation with selected motor and health-related fitness parameters.	BMI was calculated, and International Obesity Task Force (IOTF) cut-offs were used to classify children weight’s status.	A total of 14.8% of the participants were characterized as obese, without differences between genders. Girls (*n* = 328): Obese = 13.4% (*n* = 44).
Tambalis et al. (2011) [72]	Greece	Cross-sectional study	725,163 children 8 to 9 years old attending nearly all schools of primary education in Greece: 51% boys (*n* = 370,901) and 49% girls (*n* = 354,262).	To compare 12-year (1997–2008) trends in the distribution of body mass index (BMI) status and physical fitness test performances among 8–9-year-old Greek children living in rural and urban areas.	BMI was calculated, and International Obesity Task Force (IOTF) cut-offs were used to classify the children’s weight status.	Although the prevalence of obese boys and girls was similar between rural and urban areas in 1997, in the last year of the investigated period, the prevalence of obesity was greater in rural areas, in both boys and girls. In exploring the distribution of childhood obesity in the latest year by prefecture, some extensive areas of high rates, located primarily in the Aegean Sea and North Greece, have been found. Boys born in 1997: Urban: 19.7% overweight and 8.1% obese. Rural: 17.7% overweight and 8.2% obese. Boys born in 2008: Urban: 25.5% overweight and12.4% obese. Rural: 23.6% overweight; 14.1% obese. Girls born in 1997: Urban: 20.2% overweight and 7.2% obese Rural: 19.4% overweight and 7.0% obese. Girls born in 2008: Urban: 29.5% overweight and 11.3% obese. Rural: 28.6% overweight and 13.0% obese.
Magkos et al. (2006) [73]	Greece (county of Iraklio, Crete)	Epidemiological study	A total of 204 and 106 boys 9 years old, 163 and 274 boys 12 years old, and 161 and 240 boys 15 years old were randomly recruited in 1982 and 2002.	To examine the relationship between age and 20-year changes in the anthropometric characteristics of Greek boys.	BMI was calculated, and International Obesity Task Force (IOTF) cut-offs were used to classify the children’s weight status.	With respect to individual age groups, differences arose only among the 9- and 12-year-olds, in whom the prevalence of obesity more than tripled from 1982 to 2002. In 15-year-old boys, however, no significant differences could be observed.
Pavlidou et al. (2023) [74]	Greece	Cross-sectional study	5198 children aged 2–5 years (and their paired mothers, who were selected 2–5 years postpartum). Mean children’s age (±sd) = 4.1 ± 1.2 years (range: 2.0–5.5 years). Children’s gender, 49.3% = male and 50.7% = female.	To evaluate potential associations between women’s pre-pregnancy excess body weight and childhood anthropometric characteristics, as well as perinatal and postnatal outcomes.	BMI was calculated, and International Obesity Task Force (IOTF) cut-offs were used to classify the children’s weight status.	A total of 7.9% of the children were affected by obesity.
Chatzinikola et al. (2023) [75]	Greece (Rhodes)	Cross-sectional study	227 students (from one high school in the city of Rhodes, and the fifth high school of Rhodes), aged from 11 to 15 years old: 103 boys (45.4%) and 124 girls (54.6%). Mean age (years): total = 13 (12–14); boys = 13 (13–14); girls = 12 (12–14).	To investigate the adherence to the Mediterranean diet of adolescents that attend a high school on a Mediterranean island in the city of Rhodes, Greece, during the lockdown due to the COVID-19 pandemic.	BMI was calculated, and both International Obesity Task Force (IOTF) and WHO cut-offs were used to classify the children’s weight status.	Obesity (using IOTF cutoffs) = 5.3%; Obesity (using WHO cutoffs) = 8.8%.
Pappa et al. (2022) [76]	Greece	Cross-sectional study	190 primary schoolchildren, aged 6–12 years.	Τo capture the prevalence of childhood obesity and to assess its risk factors.	BMI was calculated, and International Obesity Task Force (IOTF) cut-offs were used to classify the children’s weight status.	Obesity prevalence = 10.5%. An increase in obesity was observed at 6–10 years, and a difference between the sexes was apparent, with higher rates of obesity observed in boys.
Makri et al. (2022) [77]	Greece (Attica and Thessaloniki; other regions not specified)	Cross-sectional study	Imputed data sample: 3816 adolescent students, a nationally representative sample of 11, 13, and 15-year-old students selected from the 2018 Greek arm of the HBSC study data. Gender: boys: 1898 (49.7%); girls: 1918 (50.3%). Age group: 11-year-olds: 1216 (31.9%); 13-year-olds: 1299 (34.0%); 15-year-olds: 1301 (34.1%); Region: Attica: 1427 (37.4%); Thessaloniki: 557 (14.6%); other: 1832 (48.0%); Place of birth: Greece: 3675 (96.3%); other: 141 (3.7%); Grade: Sixth: 1241 (32.5%); eight: 1307 (34.3%); tenth: 1268 (33.2%). The prevalence of obesity has also been calculated for the complete data sample (*n* = 3366), which is the sample of children with complete data available.	To estimate the prevalence of overweight and obesity in a nationally representative sample of adolescents aged 11-, 13-, and 15 years old living in Greece during 2018 and to further explore its association with diet-related behaviors and habits.	BMI was calculated, and International Obesity Task Force (IOTF) cut-offs were used to classify the children’s weight status.	In the total sample, the prevalence of obesity was 5.3%; it was 7.3% among boys and 3.4% among girls. In the complete data sample (*n* = 3366), the prevalence of obesity was 5.2%: 6.9% among boys and 3.6% among girls. Obesity: All ages (*n* = 3816): total: 5.3%; boys: 7.3%; girls: 3.4%. 11-year-olds (*n* = 1216): total: 5.7%; boys: 7.2%; girls: 4.2%. 13-year-olds (*n* = 1299): total: 4.9%; boys: 6.6%; girls: 3.4%. 15-year-olds (*n* = 1301): total: 5.4%; boys: 8.1%; girls: 2.6%.
Kostopoulou et al. (2021) [78]	Greece (Western Greece: Achaia, Ilia and Aitoloakarnania)	Cross-sectional study	3504 children aged 10–16 years, representing 10.2% of the children with the respective age range in the region of Western Greece. Those attending the fifth and sixth grades of primary schools, 10–12 years old, and the first, second, and third grades of secondary schools, 13–16 years old. Mean age in years ± sd (age range): 12.8 ± 1.4 (10–16). Boys = 1759 (50.2%). Place of residence: Urban (≥2.000 residents) = 2295 (65.5%) and rural (<2.000 residents) = 1209 (34.5%)	To evaluate the prevalence of overweight, obesity, and central adiposity through anthropometric and body composition parameters in a large sample of children and adolescents from Western Greece and to determine its cross-sectional association with sociodemographic and lifestyle factors, presumably related to overweight and obesity in children.	Obesity was defined using the Centers for Disease Control and Prevention (CDC) and the International Obesity Taskforce (IOTF) criteria.	A total of 12.1% had obesity according to the CDC criteria, whereas based on the IOTF criteria, 7.2% had obesity. Using the CDC criteria, 15.6% of the boys were obese as compared to 8.6% of the girls; using the IOTF criteria, 9.5% of the boys were obese as compared to 4.9% of the girls.
Spinelli et al. (2021) [79]	Multicenter	Cross-sectional study	(COSI 2015–2017) Countries could select one or more of the following age groups: 6.0–6.9, 7.0–7.9, 8.0–8.9, or 9.0–9.9 years. Children enrolled in Greece: 6-year-olds = 0; 7-year-olds = 1898; 8-year-olds = 0; 9-year-olds = 1874	To assess the weight status of primary school-aged children living in 36 countries and to compare the burden of childhood overweight, obesity, and thinness in different areas of the WHO European Region—namely Northern, Eastern, and Southern Europe and Central Asia.	BMI was calculated, and both International Obesity Task Force (IOTF) and WHO cut-offs were used to classify the children’s weight status.	Obesity (WHO criteria) COSI round 4 (2015–2017): Total: boys = 20.1%; girls = 14.3%. Boys: 7-year-olds = 20.1%; 9-year-olds = 21.8%. Girls: 7-year-olds = 14.3%; 9-year-old = 12.2%.
Kanellopoulou et al. (2021) [80]	Greece (Attica, Crete, and Peloponnese)	Cross-sectional study	1700 schoolchildren (10–12 years old). Boys = 781 (45.9%). Girls = 919 (54.1%).	To investigate the association between children’s adherence to a posteriori-defined dietary patterns and obesity status, in relation to weight perception, in Greece.	BMI was calculated, and International Obesity Task Force (IOTF) cut-offs were used to classify the children’s weight status.	A total of 87 participants were obese --> obesity rate = 5.1%.
Tsekoura et al. (2021) [81]	Greece (Western Greece)	Cross-sectional study	3504 students 10–16 years old; mean age (years ± SD): 12.8 ± 1.4. Males: 50.2% (*n* = 1759) and females: 49.8% (*n* = 1745). Children of primary (fifth and sixth graders) and secondary education were included in the study.	To identify the risk for developing eating disorders in children and adolescents with normal and excessive body weight.	BMI was calculated, and International Obesity Task Force (IOTF) cut-offs were used to classify the children’s weight status.	Obesity = 12.1% (*n* = 424).
Champilomati et al. (2020) [82]	Greece (metropolitan Athens area and Heraklion city area, Crete)	Cross-sectional study	1728 children (785 males), aged 10–12 years of age, attending the fifth and sixth grades of primary school.	To examine the different types of foods preferred for breakfast during childhood and their association with the development of obesity.	BMI was calculated, and International Obesity Task Force (IOTF) cut-offs were used to classify the children’s weight status.	Obesity: males = 5.3%; females = 3.1%. Mean age of obese children (years ± sd) = 11 ± 10.7. A total of 52.3% of obese children were boys.
Poulimeneas et al. (2017) [83]	Greece	Cross-sectional study	172 adolescents aged 10–15 years old. Boys = 95 (55.2%), mean age (years ± sd): 12.2 ± 1.4, and residence (urban/rural): 38/57; Girls = 77, mean age (years ± sd): 12.4 ± 1.5, and residence (urban/rural): 24/53.	To investigate the cross-correlates of pocket money on diet quality and the weight status of Greek youngsters.	BMI was calculated, and International Obesity Task Force (IOTF) cut-offs were used to classify the children’s weight status.	Obesity: total = 4.1%; boys = 6.3% (*n* = 6); girls = 1.3% (*n* = 1).
Nika et al. (2019) [84]	Greece (Kastoria)	Cross-sectional study	2832 students (children and adolescents) aged 6–18 years old. Mean age (years ± sd): 11.22 ± 3.16. Sex (male/female): 1413/1419 (49.9%/50.1%).	To assess the prevalence of high BP levels according to the European Society of Hypertension (ESH) 2016 guidelines [115] and to investigate risk factors for BP elevation in childhood and adolescence.	BMI was calculated, and International Obesity Task Force (IOTF) cut-offs were used to classify the children’s weight status.	Obesity = 8.5% (*n* = 240). There was no difference in the prevalence of obesity between urban and rural areas, or among seasons of screening.
Spinelli et al. (2019) [85]	Multicenter	Cross-sectional study	(COSI 2005–2013) Countries could select one or more of the following age groups: 6.0–6.9, 7.0–7.9, 8.0–8.9, or 9.0–9.9 years. Children enrolled in Greece: 10,616, Mean age (years): round 1 = 7.9 and round 2 = 7.9.	To assess the prevalence of severe obesity in 6- to 9-year-old schoolchildren from 21 countries of the WHO European Region, which participated in the WHO European Childhood Obesity Surveillance Initiative (COSI) data collection process between 2007 and 2013.	BMI was calculated, and International Obesity Task Force (IOTF) cut-offs were used to classify the children’s weight status.	Prevalence of severe obesity—according to the WHO definition: boys = 7.2%, girls = 2.4%, and total = 4.8%; Prevalence of severe obesity— according to IOTF definition: boys = 2.8%, girls = 2.7%, and total = 2.8%.
Zhao et al. (2019) [86]	Multicenter	Cross-sectional study	3497 children and adolescents aged 6–17 years for all countries.	To examine the association between “metabolically healthy obesity” (MHO) and high cIMT in children and adolescents using population-based data from five countries.	BMI was calculated, and International Obesity Task Force (IOTF) cut-offs were used to classify the children’s weight status.	Greece Total: metabolically healthy obese *n* = 11 (boys = 54.6%) and metabolically unhealthy obese *n* = 43 (boys = 60.5%) Age year (sd): metabolically healthy obese = 12.6 (2.8) and metabolically unhealthy obese = 13.3 (1.9).
Papoutsakis et al. (2018) [87]	Greece (Penteli, Athens)	Cross-sectional study (case–control)	514 children (217 asthma cases and 297 healthy controls) aged 5–11 years recruited between November 2007 and September 2010 at the Department of Allergy-Pneumonology, Penteli Children’s Hospital, Penteli, Greece, and two municipal multi-clinic centers in Galatsi and Pefki, in the greater area of Athens, Greece. Asthma cases: mean age = 7.7 ± 1.9 and male gender = 133 (61%). Controls: mean age = 7.6 ± 1.8 and male gender = 164 (55%).	To calculate an obesity-preventive lifestyle score comprising eating and physical activity behaviors to assess adherence to pediatric obesity prevention guidelines and evaluate the association of this obesity-preventive lifestyle score on asthma in children.	BMI was calculated, and International Obesity Task Force (IOTF) cut-offs were used to classify the children’s weight status.	Obesity = 11.3%.
Katsa et al. (2018) [88]	Greece (Sparta)	Cross-sectional study	480 schoolchildren aged 5 to 12 years. Sex: boys 46% (*n* = 223) and girls 54% (*n* = 257). Age > 9: 28.5%; 49.8% boys (*n* = 111) and 43.6% girls (*n* = 112).	To study the prevalence of MTS in healthy children aged 5 to 12 years old living in Sparta, Greece.	Obesity defined using McCarthy et al.’s (2001) [116] criteria based on age- and sex-specific percentiles.	A total of 13.33% of the children are obese (BMI ≥ 95%)
Koulouvaris et al. (2018) [89]	Greece (the 18 most remote and isolated islands of the Aegean Sea in Greece)	Cross-sectional study	463 children, aged 5–12 years, attending 25 public schools. Age group 5–6 years: Mean age male = 5.8 ± 0.38 and female = 5.8 ± 0.39. Age group 7–8 years: mean age male = 7.6 ± 0.50 and female = 7.5 ± 0.50. Age group 9–10 years: mean age male = 9.4 ± 0.49 and female = 9.5 ± 0.51. Age group 11–12 years: mean age male = 11.5 ± 0.50 and female = 11.9 ± 0.75.	First, to assess the prevalence of childhood obesity in 18 remote and isolated Greek islands and, second, to examine the association between BMI and physical fitness indices in children aged 5–12 years.	BMI was calculated, and the World Health Organization (WHO) norms were used to classify the children’s weight status.	The prevalences of obese boys was 23.8%, and for obese girls it was 13.2%. The prevalence of obese children increased progressively with age in both genders with no differences between them.
Tambalis et al. (2018) [90]	Greece	Cross-sectional study	336,014 children (51% boys and 49% girls) aged 4 to 17 years old from pre-elementary (4- to 5-year-olds), elementary (6- to 11-year-olds), and middle (12- to 17-year-olds) public and private schools (almost 40% of all schools in Greece).	The aim of the present study is to examine the prevalence of the total and central obesity groups among 4- to 17-year-old children and adolescents as a basis for effective prevention strategies and to investigate whether there is an association between several anthropometric and lifestyle factors and total/central obesity.	BMI was calculated, and International Obesity Task Force (IOTF) cut-offs were used to classify the children’s weight status.	Obesity prevalence: Boys: 4 years = 5.8%; 5 years = 8.5%; 6 years = 8.9%; 7 years = 9.2%; 8 years = 10.2%; 9 years = 10.7%; 10 years = 9.0%; 11 years = 8.8%; 12 years = 8.2%; 13 years = 8.1%; 14 years = 8.1%; 15 years = 6.8%; 16 years = 6.7%; 17 years = 6.5%. GIRLS: 4 years = 5.7%; 5 years = 8.3%; 6 years = 9.4%; 7 years = 9.2%; 8 years = 9.7%; 9 years = 9.0%; 10 years = 7.8%; 11 years = 6.4%; 12 years = 5.2%; 13 years = 4.3%; 14 years = 4.3%; 15 years = 3.9%; 16 years = 6.37%; 17 years = 4.3%. Obese girls decreased between the 4-year and 17-year groups and presented lower proportions at the age of 17 years old. In adolescence, obese rates were decreasing in both genders.
Kleanthous et al. (2016) [91]	Greece (West Attica and Athens)	Epidemiological study	1327 schoolchildren (702 boys and 625 girls) who took part in the first and last measurements during the 2.5-year study period. The children were in first grade (aged 6–7), fourth grade (aged 9–10), seventh grade (aged 12–13), and tenth grade (aged 15–16). NOVEMBER 2009	To examine the body weight status changes in schoolchildren in the Greater Athens area over a period of two and a half years (from November 2009 to May 2012) in the midst of the economic crisis.	Overweight, obesity, and underweight were defined according to the International Obesity Task Force (IOTF).	During the 2.5-year study period, there was a decrease in the prevalence of overweight and obesity. NOVEMBER 2009 Boy obesity, *n* (%): first grade: 27 (17.5%); second grade: 15 (9.7%); third grade: 23 (9.7%); fourth grade: 13 (8.4%). Girl obesity, *n* (%): first grade: 28 (21.4%); second grade: 17 (9.8%); third grade: 7 (4.0%); fourth grade: 5 (3.4%). May 2012 boy obesity, *n* (%): first grade: 24 (15.5%); second grade: 10 (6.4%); third grade: 20 (8.4%); fourth grade: 6 (3.9%). Girl obesity, *n* (%): first grade: 39 (29.8%); second grade: 40 (23%); third grade: 31 (17.9%); fourth grade: 17 (11.5%).
Hassapidou et al. (2015) [92]	Greece (Thessaloniki)	Cross-sectional study	1006 preschool children: 529 boys and 477 girls aged 2.0–6.0 years old (mean age 3.94 ± 0.87 years).	To assess overweight and obesity status in a sample of preschoolers 2–6 years old in Thessaloniki.	BMI was calculated, and the children’s weight status was defined using the IOTF, WHO, and CDC criteria.	Prevalence of obesity
Sourani et al. (2015) [93]	Greece (Tinos)	Cross-sectional study	352 children and adolescent from Tinos island aged 6–11 years (mean age: 8.5 ± 1.7 years). The majority (*n* = 192; 54.5%) of the study population consisted of boys (girls = 160). Moreover, less than one-fifth (*n* = 65; 18.5%) of the study population was of immigrant origin. Also, 6–9.9 years were classified as children, and those between 10 and 11 years were classified as preadolescents (young adolescents). Children (6–9.9 years old) (*n* = 227; 64.5%); total boys = 120; total girls = 107	To evaluate the prevalence of obesity, as well as visceral obesity, in children and young adolescents in the Greek island of Tinos.	BMI was calculated, and the children’s weight status was defined using the IOTF, WHO, and CDC criteria.	The prevalence of obesity among the study population children was 8.2% (*n* = 29). Among boys, the prevalence of obesity was 8.3% (*n* = 16); similarly, among girls the prevalence of obesity was 8.1% (*n* = 13). Approximately one-tenth of the children (*n* = 23; 10.1%) were obese. Among young adolescents the prevalence of obesity was approximately one-tenth (*n* = 6, 4.8%). No difference in terms of the prevalence of obesity (*p* = 0.148) between the two age groups was found; moreover, no differences were found with respect to the frequency of obesity between genders. Among Greek participants the prevalence of obesity was 8.71% (mean age: 7.7 ± 1.6), while among foreign immigrants it was 6.15% (mean age: 6.8 ± 1.5). In the Greek population a significant age difference (*p* = 0.001) among the three groups (normal weight, overweight, and obese) was observed, with the obese group being the youngest ones. This difference did not reach statistical significance among the immigrants. Moreover, among the Greeks, the obese participants were the tallest. Mean age of the total obese group (*n* = 29) = 7.6 ± 1.6. Obesity in (%) boys: 6 Y: 19.8; 7 Y: 20.6; 8 Y: 21.6; 9 Y: 22.8; 10 Y: 24.0; 11 Y: 25.1. Obesity (%) in girls: 6 Y: 19.7; 7 Y: 20.5; 8 Y: 21.6; 9 Y: 22.8; 10 Y: 24.1; 11 Y: 25.4.
Karachaliou et al. (2015) [94]	Greece (Heraklion, Crete)	Longitudinal study (cohort study)	595 mother–child pairs (contacts with the mothers were at 24 weeks of gestation, at birth, and at 8–10 weeks after delivery, and for the child’s follow-up, it was at the 9^th^ and 18th months, as well as at 4 years of age).	To examine the association of GWG (total and trimester-specific) with offspring birth weight, postnatal growth, obesity, and a range of cardio-metabolic risk factors at 4 years of age (waist circumference, skinfolds, blood pressure, lipids, adiponectin, leptin, and *C*-reactive protein) in the “Rhea” pregnancy cohort in Crete, Greece.	BMI was calculated, and International Obesity Task Force (IOTF) cut-offs were used to classify the children’s weight status.	The prevalence of obesity was 1% at 2 years of age, 3% at 3 years, and 4% at 4 years of age, respectively.
Giravalaki et al. (2014) [95]	Greece (Heraklion, Crete)	Cross-sectional study	2011: The study population comprised elementary (sixth grade, Minoa Pediados) and high school students. The total number of participants was 66 (30 girls (45.5%) and 36 boys (54.5%), with an average age of 13.6 years (standard deviation = 0.9). 1989: The study population comprised 150 adolescents, 85 girls (56.7%) and 65 boys (43.3%), from Agia Varvara high school; mean age = 13.1 years (standard deviation = 0.62).	To investigate all the important factors that contribute to the development of MetS as well as to explore the changes in these factors over time by comparing adolescent populations from rural areas of Heraklion, Crete, for the years 2011 and 1989.	BMI was calculated, and International Obesity Task Force (IOTF) cut-offs were used to classify the children’s weight status.	The percentage of obese adolescents in 2011 was 21.2% compared to only 1.3% in 1989. While in 1989 no girl was found to be obese; in 2011, 23.3% of the girls were obese. Prevalence of obesity, *n* (%) girls + boys: 2011 = 14 (21.2) and 1989 = 2 (1.3). girls: 2011 = 7 (23.3) and 1989 = 0 (0); boys: 2011 = 7 (19.4) and 1989 = 2 (3.1).
Manios et al. (2013) [96]	“Greece (Attica, Aitoloakarnania, Thessaloniki and Iraklio)	Cross-sectional study	2492 primary schoolchildren aged 9–13 years old. Boys (*n* = 1241) 9–11 years = 41.4% and 11–13 years = 58.6%, girls (*n* = 1251) 9–11 years = 41.9% and 11–13 years = 58.1%, and total (*n* = 2492): 9–11 years = 41.6 and 11–13 years = 58.4%.	To examine the association between being overweight and iron status in children and adolescents in Greece and to identify the main risk factors for ID.	BMI was calculated, and International Obesity Task Force (IOTF) cut-offs were used to classify the children’s weight status.	Obesity: total: 11.4%, boys = 13.3% (*n* = 165), and girls = 9.6% (*n* = 120) --> significantly different from boys;
Tambalis et al. (2013) [97]	Greece	Cross-sectional study	3195 schoolchildren aged 10 to 12 years attending fifth and sixth grades living in rural and urban areas.	To examine the hypothesis that Greek children living in rural areas are more overweight and obese than their urban counterparts because of lower levels of PA.	BMI was calculated, and International Obesity Task Force (IOTF) cut-offs were used to classify the children’s weight status.	Obesity rates were 1.4% higher in rural areas than in urban areas for both genders aged 10 to 12 years. Total obese (N) = 366. Rural boys (*n* = 292): mean age year (sd) = 11.0 (1.0) and obese = 12.5%;
Kollias et al. (2013) [98]	Greece (Samos island, Karlovassi province)	Cross-sectional study	448 (aged 10–18 years) apparently healthy adolescents recruited from five Karlovassi schools. Mean age: 14 ± 2.2 years. Sex: 211 boys and 237 girls.	To investigate the association between cIMT and several cardiovascular risk factors in an apparently healthy population of adolescents.	BMI was calculated, and International Obesity Task Force (IOTF) cut-offs were used to classify the children’s weight status.	Obesity prevalence = 12.7%.
Chalkias et al. (2013) [99]	Greece (Athens)	Cross-sectional study	A total of 18,296 students who were 8–9 years of age (51.05% males) in the primary schools of the Attica prefecture were selected.	This analysis addresses the following questions: (a) are living conditions and SES (socio-Economic status) heterogeneity in the study area related to ChO (childhood obesity) variations; and (b) if (a) is true, is this relationship stable across the study area.	BMI was calculated, and International Obesity Task Force (IOTF) cut-offs were used to classify the children’s weight status.	The ChO rate in the metropolitan area of Athens was 9.9% for students who were 8 and 9 years of age. The minimum and the maximum values found in different areas were 2.6 and 23.8%, respectively.
Vik et al. (2013) [100]	Multicenter	Cross-sectional study	Greek population: 1100 schoolchildren in their final years of primary education (aged 10 to 12 years).	To assess (i) the prevalence of eating breakfast, lunch, and dinner, as well as the prevalence of never watching TV during these meals among children aged 10–12 years in Europe; (ii) the relationship between these behaviors and weight status; and (iii) potential country differences and inequalities regarding gender, parental levels of education, and ethnicity in these behaviors among children aged 10–12 years in Europe.	BMI was calculated, and International Obesity Task Force (IOTF) cut-offs were used to classify the children’s weight status.	Obesity prevalence: 10%.
Antonogeorgos et al. (2011) [101]	Greece (Athens)	Cross-sectional study	700 students (323 male and 377 female) aged 10–12 years (4–6th grade), who were selected from 18 schools located in the Athens greater area.	To examine the association between extracurricular sport participation with the obesity status of children aged 10–12 years old living in an urban environment in Greece.	BMI was calculated, and International Obesity Task Force (IOTF) cut-offs were used to classify the children’s weight status.	A total of 9.4% (*n* = 24) of the boys and 8.6% (*n* = 25) of the girls were classified as obese.
Kontogianni et al. (2010) [102]	Greece (except the Ionian and Aegean islands)	Cross-sectional study	Subjects aged 3–18 years: children group = 3 to 12 years and adolescent group = 13 to 18 years. Children = 751, mean age: 7.6 ± 2.9 years, and 51% boys and 49% girls. Adolescents = 554, mean age: 15.5 ± 1.6 years, and 44% boys and 56% girls.	To identify the clustering of several eating and physical activity habits and behaviors and to explore their potential associations with body mass index (BMI) in a representative, cross-sectional sample of children and adolescents in Greece.	BMI was calculated, and International Obesity Task Force (IOTF) cut-offs were used to classify the children’s weight status.	Obese children: *n* (%) = 97 (12.9); mean age (years ± sd) = 6.6 ± 2.5. Obese adolescents: *n* (%) = 16 (2.8); mean age (years ± sd) = 16.0 ± 1.3.
Tsiouifis et al. (2009) [103]	Greece (Athens)	Cross-sectional study	A total of 498 students from the Leontio Lyceum (7–12th grade) were recruited: 304 boys (61.2%) and 194 girls (38.8%). The mean age was 14.1 ± 1.6 years (range: 12–17.9 years)	This report illustrates the 3L study’s aims, design, and methods, as well as the status of various baseline characteristics of the participants.	BMI was calculated, and International Obesity Task Force (IOTF) cut-offs were used to classify the children’s weight status.	Obesity (%) = 5.8.
Papandreou et al. (2007) [104]	Greece (Thessaloniki)	Cross-sectional study	A total of 524 healthy schoolchildren (275 boys and 249 girls) aged 6–15 year were included in the study.	The purpose of this study was to estimate the prevalence of OB and to investigate any associations between OB, blood pressure (BP), waist circumference (WC), serum homocysteine levels, and dietary intakes in a healthy pediatric population aged 6–15 years in Northern Greece.	BMI was calculated and International Obesity Task Force (IOTF) cut-offs were used to classify children weight’s status.	Obese *n* (%): males = 23 (8.4%); females = 18 (7.3%); total = 41 (7.8%).
Tokmakidis et al. (2007) [105]	Greece (Athens and North Attica)	Cross-sectional study	378 healthy elementary school pupils and 298 high school students. Elementary school pupils: sex: 52.1% girls (*n* = 197) and 47.9% boys (*n* = 181); nationality: Greek: 87.6%, Albanian: 10.3%, other: 1.6, and missing: 0.5%; and mean age: 11.4 ± 4 years. High school students: sex: 51.0% girls (*n* = 152) and 49.0% boys (*n* = 146); nationality: Greek: 94.3%, Albanian 3.0%, other 2.7%, and missing 0%; and mean age: 12.5 ± 3 years.	To examine the validity of self-reported body measures as a diagnostic method for the evaluation of overweight and obesity in Greek children and adolescents.	BMI was calculated, and International Obesity Task Force (IOTF) cut-offs were used to classify the children’s weight status.	Obesity: 9.5% (primary education = 9.5%; secondary education = 9.4%).
Papoutsakis et al. (2007) [106]	Greece (Attica)	Cross-sectional study	920 students in fifth and sixth grades; age range: 9.8–13.6 years and mean age: 11.2 ± 0.6; sex: 491 females (mean age = 11.2 ± 0.6) and 429 males (mean age = 11.2 ± 0.7).	1. A description of dietary and other lifestyle factors, as well as single polynucleotide polymorphisms associated with obesity. 2. The association of SNPs with overt obesity markers in children (e.g., weight status), with candidate sub-phenotypes of obesity (e.g., inflammation markers), and markers of susceptibility (e.g., family history). 3. The detection of significant interactions via association studies between genetic and environmental factors in childhood obesity.	BMI was calculated and International Obesity Task Force (IOTF) cut-offs were used to classify children weight’s status.	Obesity prevalence: total = 8.8%; females = 7.8%; males = 10.1%.
Stabouli et al. (2005) [107]	Greece	Cross-sectional study	93 consecutive adolescents (22 obese and 71 non-obese) aged 11–18 years, who were referred to the hypertension center of the study for the evaluation of borderline hypertension from their primary healthcare providers.	To investigate possible differences in 24 h ambulatory blood pressure between obese and non-obese adolescents, to measure internal carotid artery IMT, and to investigate early obesity-associated vascular structural changes.	BMI was calculated, and 2000 Centers for Disease Control and Prevention growth charts cut-offs were used to classify the children’s weight status.	A total of 24.0% of the subjects were obese. Obese group: mean age = 15.00 ± 2.01; males/females (%) = 16.9/6.8.

**Table 2:** Policy study table.

Author (Year) [Ref]	Country	Study Design	Population	Purpose	Key Results
Kastorini et al. (2019) [108]	Greece	Descriptive study	Not applicable	To describe the national dietary guidelines for children and adolescents as an essential policy tool.	The NDGGr include food-based recommendations, food education, and health promotion messages regarding (i) fruits; (ii) vegetables; (iii) milk and dairy products; (iv) cereals; (v) red and white meat; (vi) fish and seafood; (vii) eggs; (viii) legumes; (ix) added lipids, olives, and nuts; (x) added sugars and salt; (xi) water and beverages; and (xii) physical activity. A nutrition wheel, consisting of the ten most pivotal key messages, was developed to enhance the adoption of optimal dietary patterns and a healthy lifestyle. The NDGGr additionally provide recommendations regarding the optimal frequency and serving sizes of main meals, based on the traditional Greek diet.
Suggs et al. (2011) [109]	Multicenter	Descriptive study	Not applicable	To describe public support for policy measures to counteract childhood obesity and improve children’s diets in EU countries.	The EU public favors policies consisting of communication with parents, providing healthy nutrition instruction to children, and more physical activity in schools. The Greek public’s preferences for policy strategies include 1. improving children’s diets (1.a) information for parents: 50.3% (highest quartile), (1.b) education for children: 19.9% (third quartile), and (1.c) restrict ads: 12.8% (second quartile)); 2. reducing children’s obesity (2.a) physical activity in schools: 39.1% (third quartile), (2.b) diet and exercise education: 16.2% (second quartile), and (2.c) restrict ads: 10.3% (third quartile)); and 3. building more facilities (other policies options): >20% as their first policy choice.
Oldridge-Turner et al. (2023) [110]	Multicenter	Descriptive study	Not applicable	To describe the methods used to collect detailed, up-to-date information of currently implemented policy actions promoting physical activity with the purpose of populating the MOVING database to track the status of policy actions.	Physical activity policies in Greece: M: Make opportunities and initiatives that promote physical activity in schools and the community for sports and recreation = 4; O: Offer physical activity opportunities in the workplace and training in physical activity promotion across multiple professions = 3; V: Visualize and enact structures and surroundings that promote physical activity = 2; I: Implement transport infrastructure and opportunities that support active societies = 0; N: Normalize and increase physical activity through public communication that motivates and builds behavior change skills = 8; G: Give physical activity training, assessment, and counseling in healthcare settings = 2. Total = 19. (All of them are awaiting verification.)
Kovacs et al. (2020) [111]	Multicenter	Descriptive study	Not applicable	To provide an overview of the approaches to the regulation and improvement of kindergarten and the school food environment in 16 European Union Member States.	One of the seven policies identified is from Greece: “the Greek School Canteen Policy”. It fulfills the nine core good practice criteria. Characteristics of the Greek educational system: centralized; compulsory from 5 to 15 yrs (10 yrs); pre-school ages: nursery school (2–5 yrs) and kindergarten (5–6 yrs); school ages: primary (6–12 yrs), lower secondary (12–15 yrs), and upper secondary (15–18 yrs); and type of institution: public (93%) and private (7%). Legislative environment around school meal provision of school lunch: only in some private schools. For these schools, the Mediterranean diet pyramid-based dietary guidelines for Greece (Ministry of Health, 1999) is in use. Policies to restrict the availability of unhealthy foods and drinks in schools in vending machines and/or shops: mandatory policy. The Greek school canteen policy was established in 2004, had undergone some improvements (in 2006 and 2013), and took its final format in 2013 with effect until today (81025/2013—FEK 2135/Β/29-8-2013, with up-to-date annual modifications). The Ministry of Health established a law including a detailed list determining which products are permitted to be sold in Greek school canteens. Across all areas of school premises, both public and private, controls are carried out on a regular basis by the Regional Public Health Services of the Hellenic Food Authority (EFET). Legislations to restrict the marketing of unhealthy foods in educational premises: mandatory policy. Marketing or advertising any products that are not listed in the School Canteen Policy ((81025/2013—FEK 2135/Β/29-8-2013) is prohibited.
UNICEF and Greek Ministry of Health (2023) [112]	Greece	Not applicable	Not applicable	To mitigate the risk factors and the socio-economic disparities responsible for obesity in childhood and adolescence while also fighting the consequences of being overweight and obese that often lead to chronic diseases during adult life.	The program includes cross-sectoral interventions across the country; it is related to primary, secondary, and tertiary prevention and is targeted to children aged 0–17 years old, as well as their families. More specifically, the program aims to: - Establish a healthy diet and active behavior habits among Greek students via initiatives within the school units of all compulsory education levels (kindergarden, elementary school, middle school, and upper secondary school) with the engagement of teachers, parents, and guardians; - Encourage parents to receive personalized free clinical evaluations and consultations during scheduled visits with their pediatrician regarding the health, development, and nutritional status of their children, as well as regarding the risk factors related to childhood obesity and the ways of addressing them promptly; - Allow school-aged children and their families with pre-existing risk factors for obesity or other chronic diseases to receive free consultancy services (nutritional counseling and counseling to change health-related behaviors) in the form of remote consultation offered by qualified health professionals (nutritionists, fitness instructors, etc.); - Refer already overweight and obese school-aged children with pre-existing diseases and complications to specialized pediatric units for further follow-up by health professionals; - Establish a new research center, the European Centre Against Obesity, at the Harokopio University, with the aim of promoting research, generating ideas and policy proposals, and assuming an active role in the scientific coordination and evaluation of an action plan.

## Data Availability

This study did not generate any new data.

## Abbreviations

The following abbreviations are used in this manuscript:

WHO	World Health Organization
HBSC	Health Behaviour in School-aged Children
PICOS	Population, Intervention, Comparison, Outcomes, and Setting
BMI	Body Mass Index
UNICEF	United Nations Children’s Fund
IOTF	International Obesity Task Force
COSI	Childhood Obesity Surveillance Initiative
